# The Association among Antioxidant Enzymes, Autoantibodies, and Disease Severity Score in Systemic Lupus Erythematosus: Comparison of Neuropsychiatric and Nonneuropsychiatric Groups

**DOI:** 10.1155/2014/137231

**Published:** 2014-04-27

**Authors:** Yu-Jih Su, Tien-Tsai Cheng, Chung-Jen Chen, Wen-Chan Chiu, Wen-Neng Chang, Nai-Wen Tsai, Chia-Te Kung, Wei-Che Lin, Chih-Cheng Huang, Ya-Ting Chang, Chih-Min Su, Yi-Fang Chiang, Ben-Chung Cheng, Cheng-Hsien Lu

**Affiliations:** ^1^Department of Internal Medicine, Kaohsiung Chang Gung Memorial Hospital and Chang Gung University College of Medicine, Kaohsiung 833, Taiwan; ^2^Department of Biological Science, National Sun Yat-Sen University, Kaohsiung 80424, Taiwan; ^3^Department of Neurology, Kaohsiung Chang Gung Memorial Hospital and Chang Gung University College of Medicine, Kaohsiung 833, Taiwan; ^4^Department of Emergency Medicine, Kaohsiung Chang Gung Memorial Hospital and Chang Gung University College of Medicine, Kaohsiung 833, Taiwan; ^5^Department of Radiology, Kaohsiung Chang Gung Memorial Hospital and Chang Gung University College of Medicine, Kaohsiung 833, Taiwan

## Abstract

*Background*. Antioxidative capacity plays an important role in the severity of systemic lupus erythematosus (SLE), which is characterized by autoantibodies. This study aimed to determine the relationship among autoantibody titers, antioxidative stress reserve, and severity of SLE. *Methods*. The autoantibody titers, clinical markers, antioxidant enzyme levels, and disease activity index (SLEDAI-2k) of 32 SLE patients and 16 healthy controls were compared. We also compared both the neuropsychiatric (NPSLE) and nonneuropsychiatric (non-NPSLE) groups. *Results*. Superoxide dismutase in red blood cells was significantly lower in the SLE than in the control group. CRP levels are significant higher in SLE patients than in control group (*P* = 0.034). Among the autoantibodies, anti-U1RNP (*P* = 0.008), a-Sm (*P* = 0.027), and anti-ribosomal p (*P* = 0.028) significantly negatively correlated with glutathione levels. There has no significant correlation between SLE disease activity indexes (SLEDAI) and levels of C3, C4, and antioxidant enzymes. *Conclusions*. Erythrocyte superoxide dismutase is significantly lower in both NPSLE and non-NPSLE groups. SLE patients have both higher CRP and autoantibodies level and decreased superoxide dismutase level than the healthy control group.

## 1. Introduction

Autoimmune diseases are characterized by the deviated activation of the innate and adaptive immune systems. Autoantibodies are markers of B-cell activation in adaptive immunity. Some are diagnostic and specific, such as anti-nuclear antibody (ANA) and anti-double stranded deoxyribonucleic acid antibody (a-ds DNA) for systemic lupus erythematosus (SLE) and perinuclear anti-neutrophil cytoplasmic antibodies (p-ANCA) and cytoplasmic anti-neutrophil cytoplasmic antibodies (c-ANCA) for vasculitis.

Inflammation and increased oxidative stress are recognized in almost all patients with autoimmune diseases. The effect of oxidative stress may lead to cell apoptosis and further antigen presentation and autoantibody formation. On the other hand, host factor and disease characteristics are important factors [1, 2]. Lipid peroxidation of erythrocyte membranes by toxic oxygen-free radicals plays a major role in red blood cell hemolysis, which is common in SLE. Increased sensitivity of red blood cells (RBCs) to oxidative damage is therefore an index of antioxidant deficiency. The steady-state formation of oxidants is balanced by a similar rate of their consumption by antioxidants. This study was designed to evaluate changes in the activities of three antioxidant enzymes, including superoxide dismutase (SOD-rbc) and glutathione peroxidase (GPX-rbc) activities on RBCs, and serum glutathione levels of SLE patients.

The SOD-rbc is one of the three forms of SOD in human cytoplasm of cells. Because of the large amount of RBCs widely distributed in blood, SOD in RBCs is a major source of antioxidant against the dismutation of superoxide (O_2_^−^). The glutathione peroxidase is a group of enzymes that convert lipid hydroperoxides to alcohols and hydrogen peroxide to water and protect organism against oxidative damage. The glutathione is also an antioxidant and concentration of the reduced state of the glutathione as an indicator of antioxidant capacity.

SLE patients may suffer from skin, joint, hematologic, renal, pulmonary, and neurological diseases. Additionally, the course of the disease is highly variable between patients, with certain manifestations more common than others and the overall impact on quality of life dependent on the individual patient's circumstances and particular disease manifestations. Neuropsychiatric symptoms occur in between 30% and 40% of SLE patients, which can constitute the initial presentation, and may occur in the context of a SLE flare. The clinical features can experience acute neurological events such as seizures, cerebrovascular accidents, and delirium and psychiatric conditions including depression, anxiety, and psychosis, as well as memory loss and general cognitive decline.

To our knowledge, only several articles have focused specifically on the activities of antioxidant enzymes in peripheral blood of SLE [3–5]. Further, research into the underlying mechanisms of NPSLE and the role of antioxidant reserve was less mentioned elsewhere. This study hypothesizes that antioxidative capacity plays an important role in SLE severity. A better understanding of the pathophysiology of SLE is essential for the treatment or prevention of flareups. This study aimed to determine the relationship among levels of antioxidant enzymes and autoantibodies and severity of SLE.

## 2. Patients and Methods

### 2.1. Study Patients

This study included 32 SLE patients and 16 healthy controls. The SLE patients were followed up at the Rheumatology Outpatient Clinic between February 2012 and March 2013 and were prospectively evaluated. The diagnostic criteria for SLE were based on the 1997 revised American College of Rheumatology (ACR) classification [6]. The Institutional Review Committee on Human Research approved the study protocol and all participants provided informed consent. Disease activity was recorded according to SLE disease activity index 2000 (SLEDAI-2k) [7]. Characteristically, SLE patients can be classified into two groups, which are NPSLE and non-NPSLE groups, according to the ACR classification [8]. The health subjects, who have not any major underlying diseases or take any medicines including antioxidant agents, were enrolled as control group.

### 2.2. Blood Sampling and Assessment of Oxidative Stress

Blood samples were collected by venipuncture of forearm veins from the patients and controls.

### 2.3. Erythrocyte Assay of Antioxidant Enzymes

#### 2.3.1. Erythrocyte Superoxide Dismutase (SOD) Activity

Superoxide dismutase activity was measured in erythrocytes using a commercially available kit (Ransod, Randox Lab, Grumlin, UK) based on the method developed by McCord and Fridovich [9]. The role of SOD was to accelerate the dismutation produced during oxidative energy processes of superoxide radicals (O_2_^−^) to hydrogen peroxide and molecular oxygen. This method employed xanthine and xanthine oxidase to generate superoxide radicals that react with 2-(4-iodophenyl)-3-(4-nitrophenol)-5-phenyltetrazolium chloride to form a red formazan dye. The SOD activity was then measured by the degree of inhibition of this reaction. The assay was conducted on washed RBCs by diluting the samples to obtain 30%–60% inhibition. A standard was supplied with the kit and was diluted to provide a range of standards and a calibration curve. A standard curve was produced by plotting the percentage (%) inhibition for each standard against Log 10. The result was multiplied by the appropriate dilution factor (100) and expressed in units/liter (U/L) of whole blood.

#### 2.3.2. Erythrocyte Glutathione Peroxidase (GPX) Activity

Erythrocyte GPX activity was measured using a commercially available kit (Ransel; Randox Lab, Crumlin, UK). Heparinized whole blood samples were diluted to convert glutathione peroxidase to its reduced form. The sample was incubated for 5 min and then diluted with Drabkin's reagent to avoid false elevation due to the presence of peroxidases in human blood. The diluted sample was mixed with reagent (glutathione, glutathione reductase, and NADPH) and cumene hydroperoxide. Glutathione (GSH) was reduced by cumene hydroperoxide and then oxidized in a GPX-catalyzed reaction. In the presence of glutathione reductase (GR) and NADPH, the oxidized glutathione (GSSG) was immediately converted to the reduced form with a concomitant oxidation of NADPH to NADP+. The decrease in absorbance after 1 and 2 min at 340 nm was measured. The results were expressed in units/liter of hemolysate and multiplied by the appropriate dilution factor to obtain result in U/L of whole blood.

#### 2.3.3. Determination of Serum Glutathione Content

The ability of antioxidative defense in response to increased oxidative damage was evaluated by measuring the plasma level of total reduced glutathione as plasma glutathione physiologically free of radical scavengers. Plasma free glutathione was determined by directly reacting glutathione with 5,5-dithiobis 2-nitrobenzoic acid (DTNB) to form 5-thio-2-nitrobenzoic acid (TNB). The amount of glutathione in the sample was calculated from the absorbance using the extinction coefficient of TNB (molar extinction coefficient of TNB was 13,600 M^−1^ cm^−1^ at 412 nm).

### 2.4. Autoantibody Detection

Autoantibody titers, including IgG antibodies against dsDNA (a-dsdna), Ribosomal p (a-rib p), Ro (52/60) (a-ro), Ro 52 kDa (a-ro52), Ro 60 kDa (a-ro60), La (a-la), U1RNP (a-u1rnp), Sm (a-sm), Mi-2 (a-mi2), perinuclear anti-neutrophil cytoplasmic antibodies (p-ANCA), cytoplasmic anti-neutrophil cytoplasmic antibodies (c-ANCA), and Jo-1 (a-jo1), were detect by Thermo Scientific Phadia system. They were detected by the system according to the operations manual.

### 2.5. Statistical Analysis

Data were expressed as mean ± SD or median (interquartile range). Categorical variables were compared using Chi-square test or Fisher's exact test, as appropriate. Levels of continuous variables were logarithmically transformed to improve normality. Univariate analyses were compared using Student's *t*-test. Continuous variables among three groups (NPSLE and non-NPSLE and control groups) were compared using one-way ANOVA, followed by post hoc multiple comparison procedure. Correlation analysis was used to evaluate the relationship between level of autoantibodies and oxidative stress markers using the Spearman method. Statistical significance was set at *P* < 0.05. All statistical calculations were performed using the SAS software package, version 9.1 (2002, SAS Statistical Institute, North Carolina).

## 3. Results

### 3.1. Baseline Characteristics of the Study Patients

Thirty-two adults aged 26–58 years with SLE were enrolled. The baseline clinical characteristics and laboratory data of the patient and control groups were listed in Tables 1 and 2. There was no significant difference in age and sex. The clinical symptoms of the 32 SLE patients included neurological involvement in 15, musculoskeletal involvement in 12, renal involvement in 10, mucocutaneous involvement in five, hematologic involvement in four, vasculitis in four, and cardiorespiratory involvement in two patients. Fifteen patients with neurological involvement were classified as NPSLE, and the other 17 patients were non-NPSLE group. Their available clinical autoantibody titers and three oxidative stress markers, including glutathione, SOD-rbc, and GPX-rbc, were also listed. The results demonstrated that CRP levels are higher in non-NPSLE group as compared with control group (*P* = 0.034). The CRP levels were not significantly different between NP-SLE and non-NP-SLE groups (*P* = 0.997). The SOD-rbc levels are significantly lower in both NPSLE and non-NPSLE groups, as compared with control group (both *P* value < 0.001) (Table 1).

### 3.2. Correlation among Oxidative Stress Markers, Antibodies, and Diseases Severity Scores

There was no significant correlation between SLE disease activity indexes (SLEDAI) and levels of C3, C4, and antioxidant enzymes. Autoantibody titers, including a-u1rnp (*P* = 0.008), a-Sm (*P* = 0.027), and a-rib p (*P* = 0.028), significantly but negatively correlated with serum glutathione level (Table 3).

## 4. Discussion

Oxidative stress represents the sum of inflammation in patients, including subjective [10] and objective [11] measures. Several lines of evidence reveal that poor clinical outcome is correlated with elevated oxidative stress. Outcome measurement ranges from high-dose immunosuppressants [12, 13], prolonged steroid treatment [14], early atherosclerosis [15], insulin-resistance [16, 17], hypertension [18], proteinuria [19], and liver damage [20] to overall systemic lupus erythematosus disease activity index [21]. Previous studies have focused on antiphospholipid and autoantibodies [22, 23], whereas the present one shows another link between autoantibodies and oxidative stress.

This study has two major findings. First, superoxide dismutase in RBC is significantly lower in both NPSLE and non-NPSLE as compared with the control group. Second, the autoantibodies, including a-u1rnp (*P* = 0.008), a-Sm (*P* = 0.027), and a-rib p (*P* = 0.028), significantly negatively correlate with serum glutathione level. Clinically, these autoantibodies represent different patient subsets. For instance, a-u1rnp is a marker for mixed connective tissue disease and is associated with mild forms of SLE [24]. a-Sm and a-rib p are specific for SLE [25], while an a-rib p has been proposed as a marker of NPSLE [26].

There is a trend of lower GPx-rbc in SLE patients, but not reach statistically significance. This might be due to the fact that the clinical symptoms of the SLE patients vary from mild disease (represented by positive a-u1rnp) to neuropsychiatric involvement (represented by a-rib p). The disease pathogenesis is also heterogeneous in SLE patients; as a result, antioxidant enzyme in RBC may not be sufficient to represent the overall oxidative stress. Compartmentalized oxidative stress in either self-reactive T cells, B cells, or phagocytic cells should be further studied [13].

NPSLE is a more severe form and may be a devastating subgroup of SLE, compared to non-NPSLE [27, 28]. In our study, we show that NPSLE and non-NPSLE patients have similar antioxidant reserves and CRP levels. This might indicate that the pathogenesis of NPSLE might not depend on the traditional inflammatory pathway, which cannot be reflected on the antioxidant reserves and CRP levels or just because these patients were in the convalescent stage. The pathogenesis of NPSLE could be either antibody dependent [29, 30] or degeneration [31–33], which both show little inflammation. The SLE disease activities were not different between NP-SLE and non-NPSLE in our study, which might be due to selection bias. We collected our patients from outpatient clinic, who are mostly stabilized patients.

This study also has several limitations. First, most of the SLE patients were in the convalescent stage. Thus, there was no significant correlation between SLEDAI and C3, C4, and antioxidant enzyme levels. Second, this is a cross-sectional observational study. Several immunosuppressant drugs (e.g., steroids) or antioxidant agents (e.g., vitamin E, coenzyme Q10) could influence the levels of antioxidant enzymes and may cause potential bias in the statistical analysis. Third, the diversity of SLE patients and various disease pathogenesis could be a bias in such a cross-sectional study. Lastly, the case numbers are small. Large-scale prospective studies are warranted to evaluate the prognostic contribution of leukocyte apoptosis on clinical outcomes.

In conclusion, erythrocyte superoxide dismutase is significantly lower in both NPSLE and non-NPSLE groups. Among the autoantibodies, a-u1rnp, a-Sm, and a-rib p are significantly correlated with serum glutathione levels. Further understanding of the specific mechanisms of antioxidative capacity in SLE may lead to new strategies that will improve patient outcome.

## Figures and Tables

**Table 1:** Baseline characteristics of the SLE patients and control subjects.

	SLE		Control^*γ*^	*P* value^*α*,*β*,*γ*^
	NPSLE^*α*^, *n* = 15	Non-NPSLE^*β*^, *n* = 17	*n* = 16	
Age	44.8 ± 12.6	38.2 ± 11.8	46.1 ± 12.2	0.159
Sex				0.992
Female	13	15	14	
Male	2	2	2	
*Presented with median *(*IQR*)				
White blood cell	6700 (4900.00, 9500.00)	5500 (4500.00, 6850.00)	5500 (4425.00, 6000.00)	0.44
Granulocyte (%)	70 (59.00, 82.80)	68.6 (60.98, 80.23)	55.9 (52.00, 68.20)	0.20
Lymphocyte (%)	24 (11.60, 26.70)	22.85 (12.03, 30.73)	34.8 (24.30, 39.00)	0.10
Monocyte (%)	5.5 (3.45, 10.10)	5.45 (4.03, 7.15)	5.9 (4.60, 6.80)	0.72
Hemoglobulin	11.9 (10.90, 13.20)	12.15 (9.90, 13.28)	13.45 (12.33, 14.10)	0.10
Platelet	216 (105.00, 251.00)	235.5 (171.75, 338.00)	205.5 (180.75, 256.00)	0.71
ESR	31 (11.00, 42.00)	18 (9.25, 36.75)	12 (10.00, 1.007)	0.38
CRP	1.6 (0.52, 17.10)	4.97 (0.99, 13.33)	0.57 (0.43, 1.27)	0.034*
Creatinine	0.63 (0.51, 0.86)	0.68 (0.54, 0.84)	ND	0.73
a-dsDNA	56.4 (15.00, 199.58)	34.95 (2.50, 163.00)	ND	0.51
a-ro	0.5 (0.10, 56.00)	0.3 (0.00, 300.00)	ND	0.25
a-la	0.1 (0.00, 0.75)	0 (0.00, 300.00)	ND	0.02*
p-ANCA	0 (0.00, 0.00)	0 (0.00, 0.10)	ND	0.18
c-ANCA	0 (0.00, 0.10)	0.1 (0.00, 0.10)	ND	0.10
a-U1RNP	0.8 (0.10, 1.20)	3.65 (1.25, 84.75)	ND	0.08
a-sm	0.1 (0.01, 0.30)	0.65 (0.20, 2.90)	ND	0.29
a-rib p	0.2 (0.10, 0.50)	0.7 (0.50, 55.00)	ND	0.37
a-ro60	0.9 (0.00, 98.00)	0.2 (0.1, 300.00)	ND	0.30
a-*β*2GP1	0.01 (0.01, 0.8)	2.8 (0.01, 14)	ND	0.06
a-Cardiolipin G	2.3 (0.83, 11)	7.2 (1.4, 46)	ND	0.37
a-Cardiolipin M	0.01 (0.01, 0.45)	0.01 (0.01, 0.21)	ND	0.88
Glutathione	1.44 (1.14, 1.67)	1.2 (0.90, 1.50)	1.14 (1.01, 1.29)	0.08
SOD-rbc	1770.94 (1349.83, 1957.72)	1409.97 (1250.2, 1519.54)	2983.7 (2204.70, 3281.10)	<0.001*
GPX-rbc	51.99 (39.20, 73.90)	52.62 (36.16, 74.06)	57.1 (46.10, 66.40)	0.48

SLE, systemic lupus erythematosus; NP, neuropsychiatric; ESR, erythrocyte sediment rate; CRP, c-reactive protein; a-dsDNA, antidouble strand DNA; a-ro, anti-Ro52/60 kDa; a-la, anti-La; p-ANCA, perinuclear anti-neutrophil cytoplasmic antibodies; c-ANCA, cytoplasmic anti-neutrophil cytoplasmic antibodies; anti-U1 ribonucleoprotein; a-sm, anti-Smith; anti-rib p, antiribosomal p; a-ro60, anti-Ro 60 kDa; a-*β*2GP1, anti-*β*2 glycoprotein I; a-cardiolipin G, a-cardiolipin IgG; acardiolipin M, a-cardiolipin IgM; SOD-rbc, superoxide dismutase; GPX-rbc, glutathione peroxidase;. IQR, inter-quartile range.

^*αβ*^Patients with (*α*) or without (*β*) neuropsychiatric symptoms or signs related to SLE disease activity. ^*γ*^Normal control group. *P* value was acquired by using one-way ANOVA-Bonferroni test.

*Indicates *P* < 0.05.

**Table 2:** Clinical data of SLE patients.

	SLE		Total, *n* = 32
	NPSLE, *n* = 15	Non-NPSLE, *n* = 17	
Clinical symptoms			
Constitutional	0	2	2
Vasculitis	2	2	4
Mucocutaneous involvement	2	3	5
Neurological involvement	15	0	15
Musculoskeletal involvement	2	10	12
Cardiorespiratory involvement	2	0	2
Renal involvement	2	8	10
Hematologic involvement	2	2	4
Median (IQR) SLEDAI-2K	8 (6.00, 14.00)	5.5 (2.25, 11.75)	6 (4.00, 12.00)
Mean dosage of medication			
Prednisolone	21.59 (mg/day) (*n* = 11)	18.90 (mg/day) (*n* = 5)	20.76 (mg/day) (*n* = 16)
Hydroxychloroquine	360 (mg/day) (*n* = 10)	250 (mg/day) (*n* = 12)	300 (mg/day) (*n* = 22)
Azathioprine	50 (mg/day) (*n* = 2)	50 (mg/day) (*n* = 1)	50 (mg/day) (*n* = 3)
Mycophenolate	1080 (mg/day) (*n* = 1)	0	1080 (mg/day) (*n* = 1)
Cyclophosphamide	500 (mg/month) (*n* = 1)	0	500 (mg/month) (*n* = 1)
Cyclosporine	25 (mg/day) (*n* = 1)	50 (mg/day) (*n* = 3)	43.75 (mg/day) (*n* = 4)

**Table 3:** Correlation among oxidative stress markers, antibodies, and diseases severity scores in SLE.

Variables		Biomarker of antioxidants		
		Glutathione	SOD-rbc	GPX-rbc
SLEDAI	Correlation coefficient	−0.295	0.017	0.123
	*P* value	0.113	0.929	0.519
C3	Correlation coefficient	0.177	0.086	0.083
	*P* value	0.35	0.651	0.663
C4	Correlation coefficient	−0.08	0.073	0.008
	*P* value	0.673	0.701	0.968
a-dsDNA	Correlation coefficient	0.02	0.119	0.247
	*P* value	0.917	0.54	0.197
a-U1RNP	Correlation coefficient	−0.588**	−0.451	−0.409
	*P* value	0.008	0.053	0.147
a-Sm	Correlation coefficient	−0.506*	−0.269	−0.291
	*P* value	0.027	0.265	0.314
a-rib p	Correlation coefficient	−0.490*	−0.348	0.184
	*P* value	0.028	0.132	0.437
CRP	Correlation coefficient	0.236	−0.315	0.191
	*P* value	0.268	0.134	0.371

*P < 0.05; **P < 0.01.

*r*, correlation coefficient; SLEDAI, systemic lupus erythematosus disease activity index; C3, complement 3; C4 complement 4; a-dsDNA, anti-double strand DNA; a-rib p, antiribosomal p; a-U1RNP, anti-U1 ribonucleoprotein; CRP, c-reactive protein; SOD-rbc, superoxide dismutase activity on red blood cell; GPX-rbc, glutathione peroxidase activity on red blood cell

The a-u1rnp (*P* = 0.019) was the only autoantibody that significantly negatively correlated with glutathione peroxidase activity on red blood cell (GPX-rbc) among neuropsychiatric SLE subgroups.
